# Serological Correlate of Protection Established by Neutralizing Antibodies Differs Among Dialysis Patients with SARS-CoV-2 Variants of Concern

**DOI:** 10.3390/vaccines13050518

**Published:** 2025-05-13

**Authors:** Guy Rostoker, Stéphanie Rouanet, Myriam Merzoug, Hiba Chakaroun, Mireille Griuncelli, Christelle Loridon, Ghada Boulahia, Luc Gagnon

**Affiliations:** 1 Department of Nephrology and Dialysis, Hôpital Privé Claude Galien-Ramsay Santé, 91480 Quincy-sous-Sénart, Francemgriuncelli@free.fr (M.G.);; 2 Collège de Médecine des Hôpitaux de Paris, 75610 Paris, France; 3 StatEthic, 92300 Levallois-Perret, France; stephanie.rouanet@statethic.fr; 4 Infection Prevention and Control Registered Nurse, Hôpital Privé Claude Galien-Ramsay Santé, 91480 Quincy-sous-Sénart, France; hiba.chakaroun@ramsaysante.fr; 5 IQVIA Laboratories Vaccines, Laval, QC H7V 3S8, Canada; luc.gagnon@iqvia.com

**Keywords:** anti-spike IgG antibodies, correlate of protection, dialysis, neutralizing antibodies, SARS-CoV-2 variants of concern

## Abstract

**Background:** The 2019 coronavirus disease (COVID-19) pandemic had a severe impact on frail, end-stage kidney disease (ESKD) patients, either on dialysis or transplanted, with a high mortality rate in the early waves. Vaccination against SARS-CoV-2 with mRNA vaccines has led to reduced hospitalization and mortality rates in the general population and ESKD patients. Neutralizing antibodies (NAbs) are a valuable correlate of protection after vaccination, and IgG anti-spike antibodies are considered a surrogate marker of protection. **Methods:** This study investigated the correlates of protection brought by NAb and anti-spike IgG antibodies against SARS-CoV-2 wild-type Wuhan strain and variants of concern in a cohort of 128 French patients on dialysis after vaccination with the BNT162b2 mRNA vaccine. The correlate was assessed using Receiver Operating Characteristic curves. **Results:** The level of protection for IgG anti-spike antibodies was set at 917 BAU/mL for the original Wuhan strain and 980 BAU/mL and 1450 BAU/mL, respectively, for the Delta and Omicron BA.1 variants. **Conclusions:** The level of protection can be regularly monitored by measuring IgG anti-spike antibody concentrations to allow tailored boosters of SARS-CoV-2 vaccination in this frail and immunocompromised ESKD population.

## 1. Introduction

The 2019 novel coronavirus (COVID-19) had a significant impact on patients with advanced kidney disease, either on dialysis or with a transplant, resulting in a high mortality rate during the initial outbreaks of the pandemic [1]. The administration of mRNA vaccines against SARS-CoV-2 has contributed to a significant decline in hospitalization and mortality rates within the general population and the end-stage kidney disease (ESKD) population, especially those on dialysis [1,2].

The vaccine response in the general population is best appreciated in the month following the second dose by measuring neutralizing antibodies (NAbs) according to the Food and Drug Administration (FDA) in the USA and the European Medicine Agency (EMA) in the European Union, and was considered, as early as 2022, to be a valuable correlate of protection. This was later confirmed with SARS-CoV-2 variants of interest [2,3,4]. The immunological response to full vaccination, evaluated by IgG anti-spike antibody titers (used as a surrogate marker of NAb), has been used to monitor protection in ESKD patients on dialysis and renal transplant patients, and for making decisions about boosters or the use of protective monoclonal antibodies in patients with declining antibody titers [1]. Correlates of serological protection against symptomatic infection, hospitalization, and death due to Omicron variants have been determined recently in epidemiological studies using IgG anti-spike antibodies in both ESKD patients and the general population [5,6]. The current study aimed to analyze the correlates of protection brought by NAb and anti-spike IgG antibodies against SARS-CoV-2 wild-type Wuhan strain and variants of concern in ESKD patients on dialysis.

## 2. Materials and Methods

### 2.1. Patients and Study Design

This retrospective serological study examined the quality and determinants of the vaccine response within one to three months following full vaccination with two or three doses of the Pfizer original BNT162b2 mRNA vaccine. This study was conducted between January 2021 and December 2021, coinciding with the initial availability of the Pfizer original BNT162b2 mRNA vaccine in France.

#### 2.1.1. Study Population

The present study encompassed 128 patients treated at the dialysis center of the private Claude Galien Hospital (Quincy-sous-Sénart, France). Of the 126 patients treated by hemodialysis, 124 underwent three dialysis sessions per week, with treatment modalities including classical hemodialysis, hemodiafiltration, and expanded hemodialysis (HDx). The remaining two patients were treated by peritoneal dialysis (PD).

The inclusion criteria encompassed a male or female subject aged 18 years or older who was undergoing hemodialysis or PD at the dialysis center of Claude Galien Hospital. The study population was further required to have received a primary dose of a vaccine against SARS-CoV-2. Informed consent and active participation in this study were prerequisites for subject enrollment. Additionally, subjects must have had affiliations with or been beneficiaries of a social security scheme.

The exclusion criteria included the following patient categories: those with contraindications to vaccination; those not vaccinated; those who had received any vaccine (other than the SARS-CoV-2 vaccine) within the previous three weeks; those who were protected (adult under guardianship, curatorship, or other legal protection and deprived of liberty by judicial or administrative decision); and pregnant women.

Of note, in the year 2021, 166 patients were treated by chronic dialysis at Claude Galien Hospital, of whom 38 (23%) were excluded for the following reasons: 6 (3.6%) for vaccine refusal, 1 (0.6%) patient vaccinated who refused to participate in this study, 3 (1.8%) patients under judicial guardianship, 9 (5.4%) patients vaccinated before dialysis initiation, and finally 19 (11.4%) patients on dialysis for a short time (less than 6 months) in our center in 2021; in detail, 3 patients who benefited from a renal transplantation in the early part of the year, 2 who were transferred to another dialysis center, 6 patients transferred to our center at the end of 2021, and 8 who died prematurely in the first months after dialysis initiation.

This study measured NAb for the three variants in the remaining samples from the routine serological tests of the 128 patients included. It was found that 90/128 samples allowed for the detection of NAb against the Delta variant, 44/128 samples allowed for the detection of NAb against the Wuhan variant, while 43/128 samples allowed for the detection of NAb against the Omicron variant.

#### 2.1.2. Study Design

The present investigation constitutes a monocentric observational study with a retrospective analysis of health data that are typically collated as part of routine care. Longitudinal follow-up of vaccinated patients was conducted over a 1-year period.

The following data were collected: (i) levels of anti-SARS-CoV-2 antibodies; (ii) demographic characteristics: age (in years), sex, how long they have been on dialysis (in months), and type of dialysis: traditional hemodialysis on polysulphone membrane, hemodiafiltration, hemodialysis on adsorbent membrane (PMMA, AN69), HDx, PD; (iii) presence of diabetes mellitus; (iv) Charlson Comorbidity Index (CCI), modified according to age; (v) biomarkers measured routinely for inflammation; and (vi) nutritional status, treatment of anemia: the amount of iron that was given intravenously (IV) (expressed as milligrams per month) received in the year before the first vaccine dose and the amount of erythropoietin (ARANESP/Darbepoetin) received (expressed as μg/week) in the year before the first vaccine dose.

#### 2.1.3. Study Aims

The main goal of this study was to describe how the immune system of dialysis patients responded to the Pfizer BNT162b2 mRNA vaccine against SARS-CoV-2. This study looked at the immune response of dialysis patients one to three months after they received the vaccine. The patients received either two or three doses of the vaccine.

The secondary objective of the present study was to determine the levels and characteristics of neutralizing antibodies following vaccination, in conjunction with the serologic correlate of protection, in this group of dialysis patients. This investigation focused on the Delta and Omicron variants of concern in comparison to the original Wuhan strain, with a correspondence to anti-spike antibodies, a serological marker used in routine practice.

### 2.2. Determination of IgG Anti-SARS-CoV-2 Antibodies

IgG anti-SARS-CoV-2 spike antibodies were determined as part of the routine medical check-ups of patients. The Cerballiance laboratory (Lisses, France) performed these check-ups. They used the chemiluminescence microparticules immunoassay (CMIA) Abbott SARS-CoV-2 IgG II Quant (ALINITY, Abbott, France). This test was performed monthly during the first three months after the second vaccine dose. After that, it was performed every three months. The results were expressed in UA/mL (arbitrary units). For this study, the results were later expressed in BAU/mL (Binding Antibody Units) using the equation BAU/mL = 1/7 UA/mL. The spike protein in the Abbott test is from the original Wuhan strain, and the test was adjusted later based on the level of specific antibodies from the WHO’s serum sample NIBSC 20/136.

### 2.3. Determination of Anti-SARS-CoV-2 Neutralizing Antibodies

NAbs against the parental Wuhan strain of SARS-CoV-2, as well as the Delta and Omicron BA.1 variants, were measured by IQVIA Laboratories (formerly NEXELIS, Laval, QC, Canada) using pseudotyped virus neutralization assays (PNAs) [7]. The samples used for this study were frozen serum specimens stored at −70 °C. These samples were stored for security according to French law for the purpose of conducting complementary viral serological assays.

Briefly, the SARS-CoV-2 PNA evaluates the level of neutralizing SARS-CoV-2 pseudovirus antibodies present in the human serum samples. Pseudotyped virus particles are prepared from a modified Vesicular Stomatitis Virus (VSVΔG) backbone and bear the spike glycoprotein of the SARS-CoV-2 from which the last nineteen (19) amino acids of the cytoplasmic tail were removed. The pseudoparticles contain a Luciferase reporter used for detection.

Serial dilutions of heat-inactivated human serum samples are prepared in 96-well transfer plates. The SARS-CoV-2 pseudoviruses are added sequentially to the serum dilutions at a target working dilution and incubated at 37 °C with 5% CO_2_ supplementation. Serum–virus complexes are then transferred onto plates, previously seeded overnight with Vero E6 cells, and incubated at 37 °C and with 5% CO_2_ supplementation. Following this incubation, the luciferase substrate is added to the cells in order to assess the level of luminescence per well. The plate(s) are then read on a luminescence plate reader. The intensity of the luminescence is quantified in relative luminescence units (RLUs) and is inversely proportional to the level of neutralizing antibodies present in the serum. The neutralizing titer of a serum sample is calculated as the reciprocal serum dilution corresponding to the 50% neutralization antibody titer (IC50) for that sample [7]. The protective threshold for NAb was established at a titer above 187 IC50, as reported by Atti et al., a member of the SIREN study group [8].

### 2.4. Statistical Analyses

Continuous variables were reported as median values and interquartile ranges [Q1–Q3]. Categorical variables were reported as numbers and percentages. Spearman’s rank-order correlation coefficients with 95% confidence intervals [95% CI] were used to assess the correlations between anti-SARS-CoV-2 NAb levels against the original Wuhan strain and the different variants (Delta strain and Omicron variant BA.1) and anti-SARS-CoV-2 Wuhan strain spike IgG levels.

We used a Receiver Operating Characteristic (ROC) curve to find the anti-SARS-CoV-2 Wuhan strain IgG threshold that matched the NAb level needed to stop 50% of infected cell cultures in a pseudotyped virus neutralization assay (with a titer chosen at ≥187 IC50 to ensure adequate protection). The maximum Youden index (sensitivity + specificity − 1) was used to determine the threshold with the best sensitivity and specificity. The results are shown as the Area Under the Curve (AUC), along with sensitivity, specificity, and their associated 95% confidence intervals [95% CIs]. We used this approach for the original Wuhan strain and the Delta and Omicron BA.1 variants. The analyses were performed using levels of anti-SARS-CoV-2 neutralizing antibodies determined in a pseudotyped virus neutralization assay and levels of anti-SARS-CoV-2 Wuhan strain IgG measured one month after the second or third vaccine doses (if applicable).

The software used for all statistical analyses was SAS version 9.4, developed by the SAS Institute (Cary, NC, USA).

## 3. Results

The demographic and clinical characteristics of the patients are shown in Table 1. Of note, blood samples for SARS-CoV-2 antibody measurement were collected after a median delay of 33 days (IQR: 32–35). This cohort can be considered as having many similarities with the whole population of dialysis patients in France and Western Europe (Table 1). The characteristics of dialysis and treatments for anemia are shown in Table 2.

**Figure 1:**
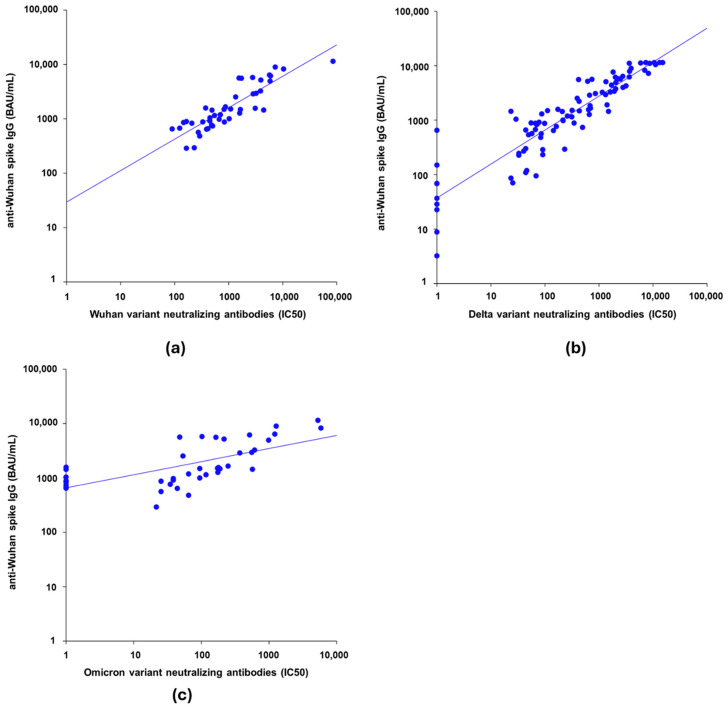
The graphs were drawn on a base-10 logarithmic scale. (**a**) Scatter plot of IgG levels against spike protein of Wuhan strain versus Wuhan variant neutralizing antibodies (N = 44). (**b**) Scatter plot of IgG levels against spike protein of Wuhan strain versus Delta variant NAb (N = 90). (**c**) Scatter plot of IgG levels against spike protein of Wuhan strain versus Omicron variant NAb (N = 43). As some NAb values were equal to 0, the data were transformed into (x + 1).

Anti-spike IgG antibodies against the Wuhan strain were highly correlated with NAbs in the pseudotyped virus neutralization assay after the second or third vaccine dose for both the Wuhan strain (rho = 0.876 [95% CI: 0.783–0.931]) (Figure 1a) and the Delta (rho = 0.928 [95% CI: 0.893–0.952]) (Figure 1b) and Omicron BA.1 variant (rho = 0.760 [95% CI: 0.595–0.863]) (Figure 1c).

**Figure 2:**
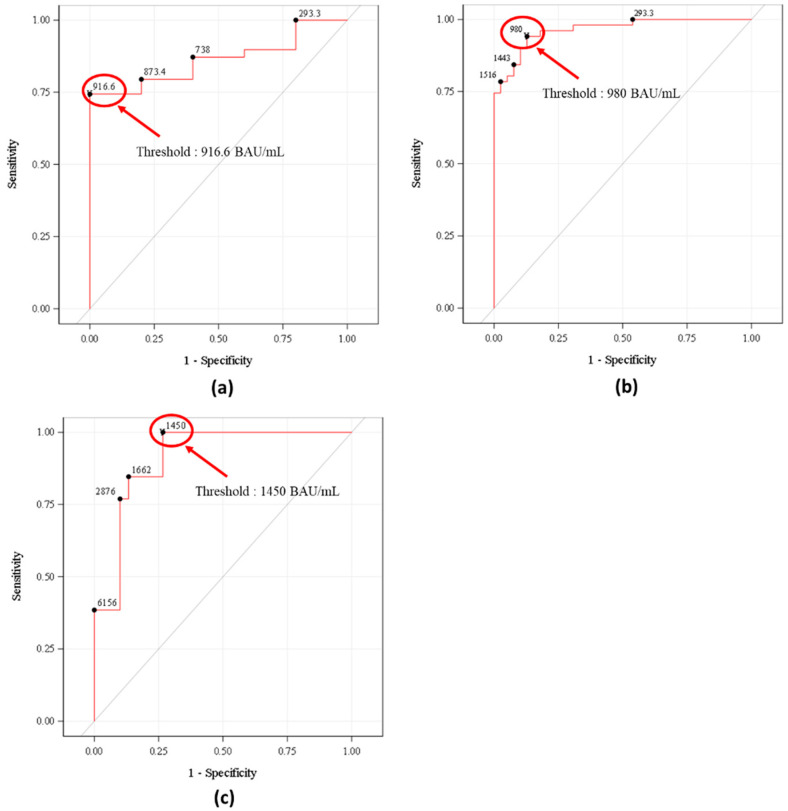
Receiver operating curves (ROCs) of IgG anti-spike protein-specific antibodies (against the Wuhan strain) for positive neutralizing antibodies at PNA against the original Wuhan strain (N = 44) (**a**), against the Delta variant (N = 90) (**b**), against the Omicron BA.1 variant (N = 43) (**c**). The predictive accuracy of Wuhan strain spike protein-specific antibodies using the chemiluminescence Abbott SARS-CoV-2 IgG II Quant assay for protective neutralizing activity (titer ≥ 187 IC50) was determined using pseudotyped virus neutralization assays against the different variants of SARS-CoV-2.

For the parental Wuhan strain, analysis was performed on 44 patients. Thirty-nine (88.6%) patients had a protective level of NAbs against the parental Wuhan strain with a titer ≥ 187 IC50 at the pseudotyped virus neutralization assay. The threshold of protection for IgG anti-spike antibodies against the Wuhan strain was set by ROC analysis at 917 BAU/mL after the second or third vaccine dose, with an AUC of 0.862 (Table 3 and Figure 2).

For the Delta strain, analysis was performed on 90 patients. Fifty-one (56.7%) patients had a protective level of NAb against the Delta strain, with an antibody titer greater than or equal to 187 IC50 at the pseudotyped virus neutralization assay. The threshold of protection for IgG anti-spike antibodies against the Wuhan strain was set by ROC analysis at 980 BAU/mL after the second or third vaccine dose, with an AUC of 0.964 (Table 3 and Figure 2).

For the Omicron variant BA.1, analysis was performed on 43 patients. Thirteen (30.2%) patients had a protective level of specific NAb against the Omicron BA.1 variant with an antibody titer ≥ 187 IC50 at the pseudotyped virus neutralization assay. The threshold of protection for IgG anti-spike antibodies against the Wuhan strain, as determined by ROC analysis, was 1450 BAU/mL after the second or third vaccine dose, with an AUC of 0.910 (Table 3 and Figure 2).

Hybrid immunity could be analyzed only in the group of patients with a measurement of NAb against the Delta variant, since 27/90 patients in this group had a prior infection with COVID-19 (and none in the groups Wuhan and Omicron). A previous infection with the virus has a significant impact on the immune response. Patients with a history of infection had a substantially higher level of NAb (2031 IC50 [Q1–Q3: 681–5884]) compared to patients without a history of infection (110 IC50 [Q1–Q3: 43–638]) (Mann–Whitney test *p* < 0.0001). Furthermore, a significantly higher percentage of patients with a prior infection (85%) had protective levels of NAb (defined as a titer equal to or greater than 187 IC50 at the pseudotyped virus neutralization assay) compared to only 44% in those without prior infection (Fisher’s exact test *p* = 0.0004).

Finally, we conducted a preliminary cost analysis of regular IgG testing against the Wuhan strain spike protein compared with a fixed booster schedule. According to the recommendation of the French Ministry of Social Affairs and Health at the time [9,10], the initial vaccination schedule for immunocompromised patients (including dialysis patients) consisted of three doses of mRNA vaccine, with booster doses repeated every three months for one year. Personalized surveillance with a threshold of 4000 UA (571 BAU/mL) was used at that time at Claude Galien Hospital and was approximately 26% more economical than the conventional regimen for the same number of patients. Moreover, a threshold of vaccination as determined in this study at the level of 6419 UA (917 BAU/mL) would have only shortened the time for the first booster dose transformed into a third dose, thereby avoiding the generation of extra costs. Furthermore, the implementation of this vaccination schedule was shown to have a significant impact on mortality rates. In 2020, 46 out of 174 patients treated by dialysis in our center contracted the SARS-CoV-2 virus, resulting in 14 fatalities (30.4% mortality rate). In contrast, in 2021, 30 out of the 166 patients at the Claude Galien dialysis center were infected by the novel coronavirus, with no observed deaths.

## 4. Discussion

This study found that the level of protection indicated by ROC analysis for IgG anti-spike antibodies against the Wuhan strain in serum samples from dialysis patients was determined to be 917 BAU/mL for the original Wuhan strain and 980 BAU/mL and 1450 BAU/mL for the Delta and Omicron BA.1 variants, respectively. It is highly probable that this finding is connected to two factors. Firstly, the immune escape of SARS-CoV-2 variants, and, secondly, the lack of acquired immunity in patients since their serum was tested in vitro after their first vaccination. This vaccination occurred after the first Wuhan wave and during the alpha variant wave, and was designed to target the Wuhan-Hu-1 strain of SARS-CoV-2, far before the delta and omicron waves.

In this study, we employed a stringent threshold of protection for neutralizing antibodies, as determined using a pseudo-virus microtitration assay (titer ≥187 IC50), aligning with the methodology established by Atti et al. [8]. Their research revealed that, among healthy UK healthcare workers, the optimal threshold for NAb was a titer of 74 IC50 in cell cultures infected with live viruses [8], which is very close to the results of 59 IC50 found in chronic kidney disease patients not on dialysis and renal transplant patients with a live virus microneutralization technique described by Knell et al. [11]. In their seminal study, Atti et al. also ensured full comparability between their pseudo-virus microneutralization technique and the gold standard live virus microneutralization technique in their cohort of healthy controls and found that a titer of 74 IC50 with the live virus microneutralization technique was equivalent to a titer of 187 IC50 with the pseudo-virus microneutralization technique. This, therefore, clarifies the selection of the aforementioned protection threshold with a titer of 187 IC50 in the present study [8].

It is noteworthy that the neutralizing threshold for SARS-CoV-2 is comparable between healthy controls and patients on dialysis and differs significantly from the classical strategy advocated in Europe and the USA for the hepatitis B vaccine. In the latter, dialysis patients exhibit a vaccine hypo-response (partly overcome with a specific vaccine schedule), and a higher level of anti-HBs antibody is required from nephrology teams to protect this vulnerable population. However, it has been demonstrated that the hypo-response status to the hepatitis B vaccine does not influence the immune response to the SARS-CoV-2 mRNA vaccine [1,12]. Furthermore, it has recently been demonstrated by our group that the magnitude of the anti-spike antibody response in dialysis patients was comparable to that of healthy controls following SARS-CoV-2 mRNA vaccination, with age, gender, and previous infection with SARS-CoV-2 taken into account [13].

Finally, a meta-analysis published in the Lancet Microbes in 2021 [4] concluded that in vitro neutralization titers remain a correlate of protection from SARS-CoV-2 variants in all populations that were studied. Furthermore, the modeling of the effects of waning immunity has the potential to predict a potential loss of protection against the variants following vaccination. Therefore, it appears that, from a scientific standpoint, it is a rational course of action to employ the same threshold of protection by neutralizing antibodies for the SARS-CoV-2 virus for healthy controls, patients on dialysis, and immunocompromised patients from diverse backgrounds.

The present study, devoted to the determination in current practice of the correlation of protection between NAb and IgG anti-spike antibodies against the Wuhan strain, complements the findings of the influence of the characteristics of dialysis patients on vaccine response recently published by our group [12]. The analysis, which used clustering, revealed three distinct clinical phenotypes among these dialysis patients in terms of immunological response. Two of these clusters included either women with a long dialysis history or patients with diabetes mellitus and a moderate history of dialysis, both of whom exhibited low levels of IgG anti-spike antibodies against the Wuhan strain. The third cluster included non-diabetic, middle-aged men with a moderate dialysis vintage and a very good serological response to vaccination. Moreover, ESA and IV iron were shown not to influence the immune response to SARS-CoV-2 vaccination [12].

These findings on the serological threshold of protection against the original Wuhan strain, namely, 917 BAU/mL, compare favourably with the optimal serological level of protection found in 2021 in the randomized efficacy trial of the ChAdOx1 nCoV-19 (AZD1222) vaccine in the UK. In this study, a vaccine efficacy of 90% against symptomatic infection with the major Alpha (B.1.1.7) variant of SARS-CoV-2 was achieved at 899 BAU/mL for IgG anti-spike antibodies against the Wuhan strain [14].

A striking difference was observed in the protective threshold for IgG anti-spike antibodies between the original Wuhan strain and the Omicron B1 variant, with an increase from 917 BAU to 1450 BAU/mL.

The threshold determined here for the BA.1 Omicron variant (1450 BAU/mL) is in line with the observation of Mink et al. during the Omicron BA.1 epidemic wave in Austria (in Vorarlberg area), showing that, in the general population, IgG anti-spike antibody levels against Wuhan strain at hospital admission were inversely correlated with in-hospital mortality, with a protective level against mortality determined at >1200 BAU/mL in patients infected with the Omicron BA.1 variant [15]. Similarly, our findings also concur with the study of Perez-Saez et al. (in the Geneva area, Switzerland), showing a protective level of IgG circulating anti-receptor-binding domain (RBD) >800 BAU/mL against Omicron infection in the general population [16], close to the threshold of 506 BAU/mL for IgG anti-RBD in dialysis patients in the USA for Omicron variants [6].

Conversely, our findings differ from a recent Japanese study performed in hemodialysis patients due to the different designs of the two studies [17]. In the Japanese study, the analysis was performed 3 weeks after the first dose of BNT162b2 vaccine, with a threshold of protection set at 18 IC50 for NAb determined with a live virus microtitration assay [17]. Takazono et al. found protection levels of IgG anti-spike antibodies at 29 BAU/mL for the original Wuhan strain and 388 BAU/mL and 3300 BAU/mL, respectively, for the Delta and Omicron BA.1 variants [17]. It is very likely that both the immune response time after vaccination and the difference in stringency of NAb thresholds explain the difference in results between these two studies [2,8,11,17].

Finally, our results fall within the suggestions of Sobhani et al. in their recent and extensive narrative review (followed by a pooled analysis of literature data) on the scope of serological correlates of protection against SARS-CoV-2, with a proposed level of IgG S1-RBD of between 1372 and 2744 BAU/mL [18].

It is highly likely that using serological thresholds in clinical practice will have the potential to significantly impact patient care. The precise adaptation of booster doses and treatments can minimize the risk of severe infections and hospitalizations in dialysis patients and other immunocompromised individuals. Furthermore, a more targeted approach to booster doses and treatments can optimize the management of health resources, including vaccines, serology, medical follow-up, and hospitalization. Moreover, an approach that incorporates individualized data can enhance patient and clinician confidence in the effectiveness of vaccination strategies. Finally, given the rapid evolution of the new SARS-CoV-2 strains and their impact on the spike protein, we strongly recommend updating the test to detect IgG antibodies to the spike protein. We urge the use of the most recent strain in lieu of the original Wuhan strain. This will facilitate, in current practice, more effective monitoring of patients with compromised immune systems, including dialysis patients.

The main limitation of our study relates to its retrospective design, which recruited all patients from a single dialysis center in a French Hospital, with patients studied after a second (good vaccine responder) or third (moderate responder to vaccination) dose, which could limit the generalizability of our findings. Therefore, definitive confirmation is warranted in larger study populations, especially multicenter studies involving dialysis patients from different countries and diverse ethnic backgrounds. These studies should also compare results across various dialysis settings, namely hospital-based centers, satellite centers, and home dialysis (including peritoneal dialysis and daily home hemodialysis). Moreover, the specific anti-T cell response against SARS-CoV-2 was not investigated in this study; this may be crucial in dialysis patients with a poor humoral vaccine response, especially with the Omicron variants [2,11,19]. Therefore, a comparison of T cell and B cell responses is essential for evaluating their respective importance in protecting the dialysis population against SARS-CoV-2.

Thus, the main takeaway of this study is that a serological follow-up of dialysis patients, based on a specified level of anti-spike antibodies (established for each variant of concern), is a viable, cost-effective, and patient-accepted clinical practice. This approach has the potential to address both hesitancy and fatigue regarding the SARS-CoV-2 vaccination in this vulnerable population.

## 5. Conclusions

The results of this study strongly suggest that the initial response to COVID-19 mRNA vaccines, 1 month after the second or third dose, in patients on dialysis, and assessed by IgG anti-spike antibodies against the Wuhan strain and neutralizing antibodies, permits a reliable determination of the correlate of protection. The level of IgG anti-spike antibodies may allow for customized boosters of the SARS-CoV-2 vaccination for frail and immunocompromised patients with ESKD on dialysis. The aim of this personalized medicine is to prevent severe and fatal SARS-CoV-2 infection in the setting of dialysis.

## Figures and Tables

**Table 1:** Demographic and clinical characteristics of dialysis patients studied for the determination of correlates of protection against SARS-CoV-2.

	All Included Patients	Group Wuhan Strain and Omicron Variant *	Group Delta Variant **
	(N = 128)	(N = 44)	(N = 90)
Age (years)			
Median	68.5	67.0	67.5
Q1–Q3	56.0–78.0	54.0–77.0	55.0–78.0
Sex, *n* (%)			
Female	49 (38.3)	15 (34.1)	33 (36.7)
Male	79 (61.7)	29 (65.9)	57 (63.3)
Weight (kg)			
Median	73.8	76.3	73.5
Q1–Q3	65.8–88.3	67.0–96.5	65.5–93.5
Body mass index (kg/m^2^)			
Median	26.0	26.5	26
Q1–Q3	22.0–30.5	24.0–33.0	22.0–32.0
Reason for kidney failure, *n* (%)			
Hypertensive nephropathy	33 (25.8)	8 (18.2)	20 (22.2)
Diabetic nephropathy	37 (28.9)	16 (36.4)	28 (31.1)
Malformation uropathy	14 (10.9)	7 (15.9)	12 (13.3)
Genetic kidney disease	13 (10.2)	7 (15.9)	10 (11.1)
Immunological or general kidney disease	19 (14.8)	2 (4.5)	11 (12.2)
Undetermined kidney disease	12 (9.4)	4 (9.1)	9 (10.0)
Diabetes mellitus, *n* (%)	54 (42.2)	21 (47.7)	37 (41.1)
Charlson Comorbidity Index (CCI)			
Median	7.0	6.5	7
Q1–Q3	5.0–8.0	5.0–7.0	5.0–8.0
CCI class, *n* (%)			
0 ≤ CCI < 4	13 (10.2)	4 (9.1)	9 (10.0)
4 ≤ CCI < 6	26 (20.3)	14 (31.8)	20 (22.2)
6 ≤ CCI < 8	48 (37.5)	17 (38.6)	35 (38.9)
CCI ≥ 8	41 (32.0)	9 (20.5)	26 (28.9)
Previous renal transplantation, *n* (%)	13 (10.2)	4 (9.1)	10 (11.1)
Previous transplantation of other organs, *n* (%)	0 (0)	0 (0)	0 (0)
Previous COVID infection prior to vaccination, *n* (%)	43 (33.6)	0 (0)	27 (30.0)
Infection with COVID after vaccination, *n* (%)	17 (13.3)	5 (11.4)	12 (13.3)
Previous viral hepatitis B, *n* (%)			
Yes	27 (21.1)	13 (29.5)	21 (23.3)
No	101 (78.9)	31 (70.5)	69 (76.7)
Viral hepatitis B vaccine response, *n* (%)			
Responder	53/101 (52.5)	17/31 (54.8)	38/69 (55.1)
Non-responder	48/101 (47.5)	14/31 (45.2)	31/69 (44.9)

* Group Wuhan strain and Omicron variant: patients were analyzed for neutralizing antibodies against the Wuhan strain and the Omicron variant. ** Group Delta variant: patients were analyzed for neutralizing antibodies against the Delta variant.

**Table 2:** Characteristics of dialysis and treatment for anemia.

	All Included Patients	Group Wuhan Strain and Omicron Variant *	Group Delta Variant **
	(N = 128)	(N = 44)	(N = 90)
Time on dialysis (months)			
Median	35.2	32.9	35.2
Q1–Q3	12.8–68.9	8.6–62.6	12.9–64.4
Dialysis method, *n* (%)			
HD	70 (55.6%)	24 (54.5)	52 (57.8)
HDF	33 (26.2%)	8 (18.2)	20 (22.2)
HDx	21 (16.7%)	10 (22.7)	16 (17.8)
PD	2 (1.6%)	2 (4.5)	2 (2.2)
Dialysis membrane, *n* (%)			
Polysulphone	69 (55.6%)	22 (52.4)	47 (53.4)
PMMA (polymethyl methacrylate)	19 (15.3%)	4 (9.5)	12 (13.6)
AN69 (polyacrylonitrile)	15 (12.1%)	6 (14.3)	13 (14.8)
MCO (medium cut-off)	21 (16.9%)	10 (23.8)	16 (18.2)
Dialysate, *n* (%)			
AX	102 (82.3%)	30 (71.4)	72 (81.8)
CX	22 (17.7%)	12 (28.6)	16 (18.2)
Vascular access, *n* (%)			
Arteriovenous fistula	84 (66.7%)	36 (85.7)	62 (70.5)
Catheter	37 (29.4%)	6 (14.3)	22 (25.0)
PTFE arteriovenous graft	5 (4.0%)	0 (0.0)	4 (4.5)
Treatment of anemia, *n* (%)			
Darbepoetin received ^†^	120/123 (97.6)	41/44 (93.2)	86/89 (96.6)
Cumulative darbepoetin dose (µg/week) ^†^			
Median	37.0	30.0	38.5
Q1–Q3	23.0–66.5	19.0–41.0	23.0–65.0
IV iron received ^†^	118/123 (95.9)	40/44 (90.9)	85/89 (95.5)
IV iron dose (mg/month) ^†^			
Median	267.0	265.0	267.0
Q1–Q3	167.0–350.0	171.0–317.0	175.0–342.0
Transfusion received ^†^	29 (22.7%)	4 (9.1%)	16 (17.8%)
Number of RBC transfused ^†^			
Median	2.0	5.5	3.0
Q1–Q3	2.0–5.0	3.0–7.5	2.0–7.5

AX: acetate dialysis bath; CX: citrate dialysis bath; HD: hemodialysis; HDF: hemodiafiltration; HDx: expanded hemodialysis; PD: peritoneal dialysis; PTFE: polytetrafluoroethylene; IV: intravenous; RBC: red blood cells. ^†^ In the year prior to vaccination. * Group Wuhan strain and Omicron variant: patients were analyzed for neutralizing antibodies against the Wuhan strain and the Omicron variant. ** Group Delta variant: patients were analyzed for neutralizing antibodies against the Delta variant.

**Table 3:** Characterization of the threshold of anti-spike IgG antibodies against the Wuhan strain using ROC analysis determined according to the level of ancestral Wuhan strain, Delta, and Omicron variants neutralizing antibodies post-second or third vaccine dose.

	Ancestral Wuhan Strain (N = 44)	Delta Variant (N = 90)	Omicron BA.1 Variant (N = 43)
Patients protected with an antibody titer ≥ 187 IC50, *n* (%) [95% CI]	39 (88.6) [76.0–95.1]	51 (56.7) [46.4–66.4]	13 (30.2) [18.6–45.1]
AUC [95% CI]	0.862 [0.741–0.982]	0.964 [0.932–0.996]	0.910 [0.825–0.995]
IgG threshold against spike protein of Wuhan strain (BAU/mL)	916.6	980	1450
Youden index	0.744	0.813	0.733
Sensitivity (%) [95% CI]	74.4 [58.9–85.4]	94.1 [84.1–98.0]	100 [77.2–100]
Specificity (%) [95% CI]	100 [56.6–100]	87.2 [73.3–94.4]	73.3 [55.6–85.8]

AUC: area under the curve. Protective antibody level if the titer is ≥187 IC50 at pseudotyped virus neutralization assay.

## Data Availability

The deidentified and anonymised data will be made available after publication upon reasonable request. Requests should be directed to rostotom@orange.fr.

## References

[1] El Karoui K., De Vriese A.S. (2022). COVID-19 in dialysis: Clinical impact, immune response, prevention and treatment. Kidney Int..

[2] Barouch D.H. (2022). COVID-19 vaccines—Immunity, Variants, Boosters. N. Engl. J. Med..

[3] Gilbert P.B., Donis R.O., Koup R.A., Fong Y., Plotkin S.A., Follmann D. (2022). COVID-19 milestone attained: A correlate of protection for vaccines. N. Engl. J. Med..

[4] Cromer D., Steain M., Reynaldi A., Schlub T.E., Wheatley A.K., Juno J.A., Kent S.J., Triccas J.A., Khoury D.S., Davenport M.P. (2021). Neutralising antibody titres as predictors of protection against SARS-CoV-2 variants and the impact of boosting: A meta-analysis. Lancet Microbe.

[5] Mink S., List W., Hoefle G., Frick M., Suessenbacher A., Winder T., Fetz C., Boesl A., Saely C.H., Drexel H. (2023). Evaluation of SARS-CoV-2 antibody levels on hospital admission as a correlate of protection against mortality. J. Intern. Med..

[6] Montez-Rath M.E., Garcia P., Han J., Cadden L., Hunsader P., Morgan C., Kerschmann R., Beyer P., Dittrich M., Block G.A. (2022). SARS-CoV-2 infection during the omicron surge among patients receiving dialysis: The role of circulating receptor-binding domain antibodies and vaccine doses. J. Am. Soc. Nephrol..

[7] Bewley K.R., Coombes N.S., Gagnon L., McInroy L., Baker N., Shaik I., St-Jean J.R., St-Amand N., Buttigieg K.R., Humphries H.E. (2021). Quantification of SARS-CoV-2 neutralizing antibody by wild-type plaque reduction neutralization, microneutralization and pseudotyped virus neutralization assays. Nat. Protoc..

[8] Atti A., Insalata F., Carr E.J., Otter A.O., Castillo-Olivares J., Wu M., Harvey R., Howell M., Chan M., Lyall J. (2022). Antibody correlates of protection from SARS-CoV-2 reinfection prior to vaccination: A nested case-control within the SIREN Study. J. Infect..

[9] Communication from the Ministry of Social Affairs and Health in Line with the Recommendations of the Vaccine Strategy Steering Committee (COSV) and the French National Authority for Health (HAS) of 6 May 2021. https://sante.gouv.fr/IMG/pdf/dgs_urgent_52_precisions_sur_la_vaccination_imd.pdf.

[10] Communication from the Ministry of Social Affairs and Health in Line with the Recommendations of the Vaccine Strategy Steering Committee (COSV) and the French National Authority for Health (HAS) of 16 September 2021. https://sante.gouv.fr/IMG/pdf/reply_dgs_urgent_90_rappel_vaccinal.pdf.

[11] Knell A.I., Böhm A.K., Jäger M., Kerschbaum J., Engl S., Rudnicki M., Buchwinkler L., Bellmann-Weiler R., Posch W., Weiss G. (2023). Virus-subtype-specific cellular and humoral immune response to a COVID-19 mRNA vaccine in chronic kidney disease patients and renal transplant recipients. Microorganisms.

[12] Rostoker G., Rouanet S., Griuncelli M., Loridon C., Boulahia G., Gagnon L. (2024). Clustering Analysis Identified Distinct Clinical Phenotypes among Hemodialysis Patients in Their Immunological Response to the BNT162b2 mRNA Vaccine against SARS-CoV-2. Vaccines.

[13] Rostoker G., Rouanet S., Merzoug M., Chakaroun H., Griuncelli M., Loridon C., Boulahia G., Gagnon L. (2025). mRNA vaccine against SARS-CoV-2 response is comparable between patients on dialysis and healthy controls after adjustment for age, gender and history of SARS-CoV-2 infection. J. Nephrol..

[14] Feng S., Phillips D.J., White T., Sayal H., Aley P.K., Bibi S., Dold C., Fuskova M., Gilbert S.C., Hirsch I. (2021). Correlates of protection against symptomatic and asymptomatic SARS-CoV-2 infection. Nat. Med..

[15] Mink S., Fraunberger P. (2023). Anti-SARS-CoV-2 antibody testing: Role and indications. J. Clin. Med..

[16] Perez-Saez J., Zaballa M.E., Lamour J., Yerly S., Dubos R., Courvoisier D.S., Villers J., Balavoine J.F., Pittet D., Kherad O. (2023). Long term anti-SARS-CoV-2 antibody kinetics and correlate of protection against Omicron BA.1/BA.2 infection. Nat. Commun..

[17] Takazono T., Ngwe Tun M.M., Funakoshi S., Morimoto S., Ota K., Torigoe K., Abe S., Muta K., Ito Y., Ashizawa N. (2023). Long-term neutralizing antibody titers after BNT162b2 vaccination in hemodialysis patients. Kidney Int. Rep..

[18] Sobhani K., Cheng S., Binder R.A., Mantis N.J., Crawford J.M., Okoye N., Braun J.G., Joung S., Wang M., Lozanski G. (2023). Clinical utility of SARS-CoV-2 serological testing and defining a correlate of protection. Vaccines.

[19] Thieme C.J., Blazquez-Navarro A., Safi L., Kaliszczyk S., Paniskaki K., Neumann I.E., Schmidt K., Stockhausen M., Hörstrup J., Cinkilic O. (2021). Impaired humoral but substantial cellular immune response to variants of concern B1.1.7 and B.1.351 in hemodialysis patients after vaccination with BNT162b2. J. Am. Soc. Nephrol..

